# Supplementary material for: Towards Preclinical Validation of Arbaclofen (R-baclofen) Treatment for 16p11.2 Deletion Syndrome
Source: bioRxiv. 2023 Sep 14:2023.05.01.538987. Preprint. [Version 2] doi: 10.1101/2023.05.01.538987 (PMC10515778; doi:10.1101/2023.05.01.538987)
Supplement: 1 [file NIHPP2023.05.01.538987V2-supplement-1.pdf]

bioRxiv preprint doi: https://doi.org/10.1101/2023.05.01.538987; this version posted September 15, 2023. The copyright holder for this preprint (which was not certified by peer review) is the author/funder, who has granted bioRxiv a license to display the preprint in perpetuity. It is made available under aCC-BY 4.0 International license.

Supplemental Figures:

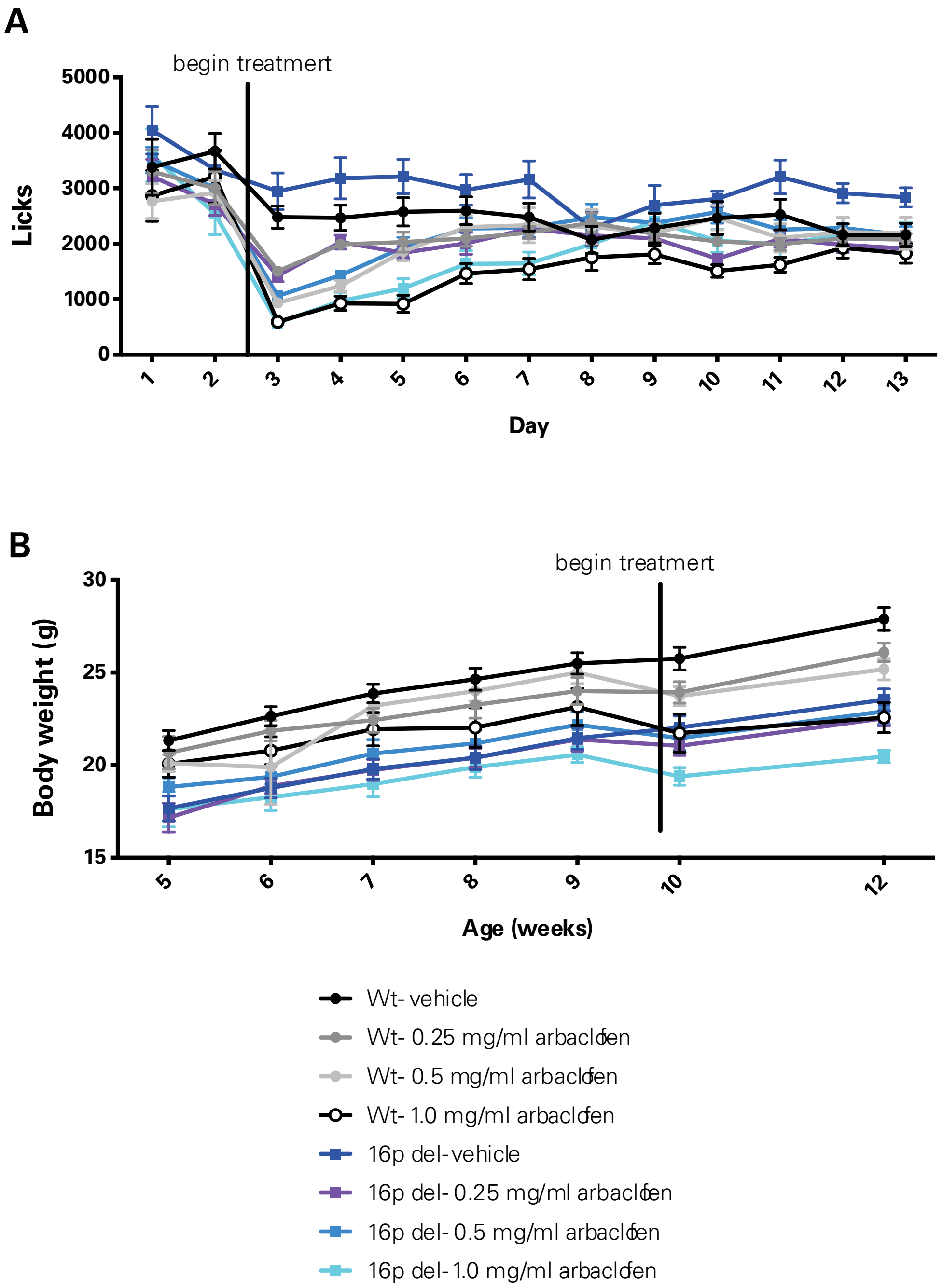

Figure S1. Arbaclofen administered in drinking water temporarily reduces drinking and body weight.

A-B. Data were collected in the Herault lab (Del6 model). **A.** Mice were housed in intellicages for 2 days prior to the start of and 12 days of arbaclofen treatment. The number of licks at water bottles for mice of each genotype is shown. Data were analyzed by 3-way RMANOVA (genotype and treatment as between-subjects factors, day as within-subjects factor). 3-way

bioRxiv preprint doi: https://doi.org/10.1101/2023.05.01.538987; this version posted September 15, 2023. The copyright holder for this preprint (which was not certified by peer review) is the author/funder, who has granted bioRxiv a license to display the preprint in perpetuity. It is made available under aCC-BY 4.0 International license.

RMANOVA: significant main effect of treatment ($F(3, 95) = 12.674$, $p = 4.88 \times 10^{-7}$), and day ($F(12, 95) = 53.323$, $p < 2 \times 10^{-16}$), no main effect of genotype ($F(1, 95) = 2.821$, $p = 0.0963$). Significant genotype x day interaction ($F(12, 95) = 1.761$, $p = 0.0499$) and treatment x day interaction ($F(36, 95) = 5.494$, $p < 2 \times 10^{-16}$. Mean +/- SEM is graphed. n = 14, 14, 13, 10, 14, 13, 16, 9.

B. Mice were weighed weekly. Body weight (g) is shown. Data were analyzed by 3-way RMANOVA (genotype and treatment as between-subjects factors, week as within-subjects factor). 3-way RMANOVA: significant main effects of genotype ($F(1, 70) = 48.829$, $p = 1.31 \times 10^{-9}$), treatment ($F(3, 70) = 3.358$, $p = 0.0236$), and week ($F(6, 70) = 141.012$, $p < 2 \times 10^{-16}$). Significant treatment x week interaction ($F(18, 70) = 4.184$, $p = 3.63 \times 10^{-8}$). Mean +/- SEM is graphed. n = 10, 10, 10, 7, 11, 11, 11, 9.

bioRxiv preprint doi: https://doi.org/10.1101/2023.05.01.538987; this version posted September 15, 2023. The copyright holder for this preprint (which was not certified by peer review) is the author/funder, who has granted bioRxiv a license to display the preprint in perpetuity. It is made available under aCC-BY 4.0 International license.

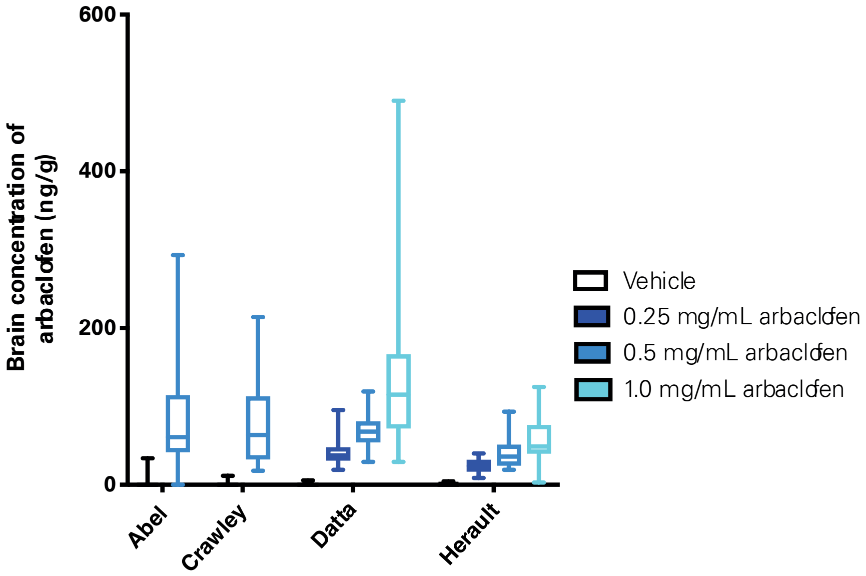

Figure S2. Arbaclofen is present in brain tissue of treated wildtype and 16p11.2 deletion model mice in dose-dependent concentrations. Brains were harvested from wildtype and 16p11.2 deletion model mice at the conclusion of behavioral studies, following 29 days of treatment. HPLC-MS/MS was used to analyze arbaclofen concentrations in frozen brain tissue, plotted here by study site. Graphs are box and whisker plots; whiskers are maximum and minimum, boxes are interquartile range, and midline is median. n = 21, 26, 34, 33, 38, 36, 41, 37, 30, 28, 29, 25

bioRxiv preprint doi: https://doi.org/10.1101/2023.05.01.538987; this version posted September 15, 2023. The copyright holder for this preprint (which was not certified by peer review) is the author/funder, who has granted bioRxiv a license to display the preprint in perpetuity. It is made available under aCC-BY 4.0 International license.

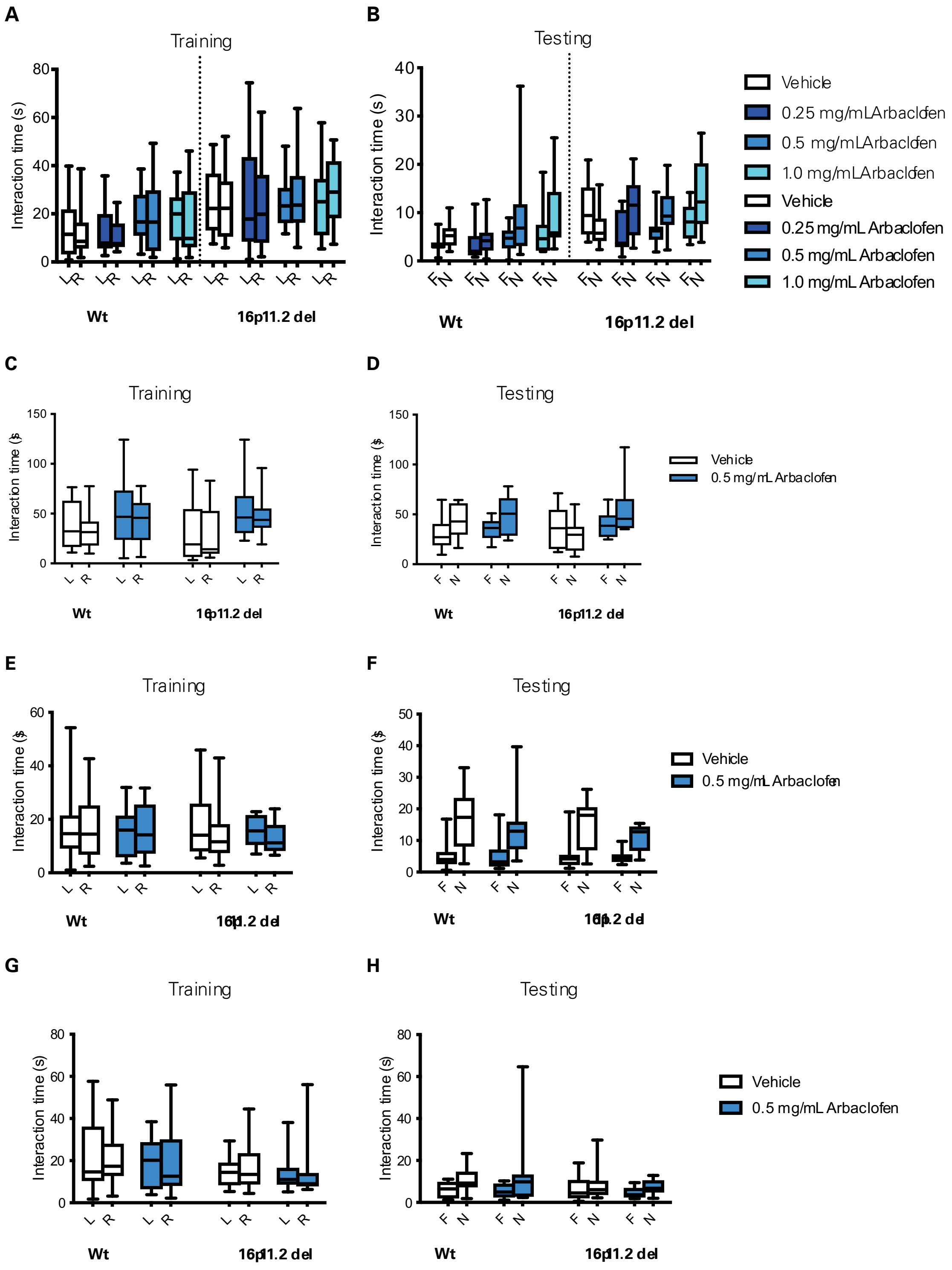

Figure S3. Arbaclofen rescues recognition memory deficits observed in two models of 16p11.2 deletion mice. Graphs are box and whisker plots; whiskers are maximum and minimum, boxes are interquartile range, and midline is median. L= left object; R= right object; F= familiar object

bioRxiv preprint doi: https://doi.org/10.1101/2023.05.01.538987; this version posted September 15, 2023. The copyright holder for this preprint (which was not certified by peer review) is the author/funder, who has granted bioRxiv a license to display the preprint in perpetuity. It is made available under aCC-BY 4.0 International license.

or location; N= novel object or location. **A-F.** During the training phase of the novel object recognition task, wt and 16p11.2 del model mice treated with vehicle or one of 3 doses of arbaclofen were allowed to interact with two identical objects. After a delay, mice were tested by exposure to one of the familiar objects and one novel object. Time spent exploring each object during training (A, C, E) and testing (B, D, F) phases is shown. Data were analyzed by 3-way ANOVA (genotype and treatment as within-subjects factors, object as within-subjects factor. **A,B:** Data collected in the Herault lab (Del6 model; 3-hour delay between training and testing). n= 14, 13, 13, 11, 13, 13, 14, 12. **A.** Training phase. 3-way RMANOVA: significant main effect of genotype ($F(1, 95) = 17.474$, $p = 6.48x\ 10^{-5}$), no significant main effect of object ($F(1, 95) = 0.182$, $p = 0.670$), treatment ($F(3, 95) = 0.805$, $p = 0.494$), or interactions. **B.** Testing phase: 3-way RMANOVA: significant main effect of genotype ($F(1, 95) = 17.234$, $p=7.21x10^{-5}$), significant main effect of object ($F(1, 95) = 25.974$, $p=1.76x10^{-6}$), no significant main effect of treatment (F((3, 95) = 2.496, p=0.0645). Significant genotype x treatment x object interaction ($F(3, 95) = 3.488$, $p = 0.01875$).

C,D: Data collected in the Abel lab (Del4 model; 24-hour delay between training and testing). n= 16, 14, 7, 12. **C.** Training phase: 3-way RMANOVA: No significant main effects of genotype ($F(1, 45) = 0.211$, $p=0.6478$), treatment ($F(1, 45) = 3.771$, $p = 0.0584$), or object ($F(1, 45) = 3.799$, $p = 0.0575$). No significant interactions.

D. Testing phase: 3-way RMANOVA: significant main effect of object ($F(1, 45) = 16.284$, $p = 0.000209$), no significant main effect of treatment ($F(1, 45) = 3.939$, $p=0.0533$) or genotype ($F(1, 45) = 0.186$, $p = 0.6681$). Trend toward genotype x treatment x object interaction ($F(1, 45) = 2.984$, $p = 0.0909$).

E,F: Data collected in the Crawley lab (Del1 model; 1-hr delay between training and testing). n= 18, 17, 14, 10

E. Training phase: 3-way RMANOVA: No significant main effects of genotype ($F(1, 55) = 0.45$, $p =0.832$), treatment ($F(1, 55) = 0.379$, $p = 0.541$), or object ($F(1, 55) = 0.704$, $p = 0.405$). No significant interactions.

F. Testing phase: 3-way RMANOVA: Significant main effect of object ($F(1, 55) = 89.456$, $p = 3.95x10\text{-}^{13}$). No significant main effects of genotype ($F(1, 55) = 0.831$, $p = 0.371$), treatment ($F(1,55)=1.911$, $p=0.172$), or significant interactions.

G-H. During the training phase of the object location memory task, wt and 16p11.2 Del1 model mice treated with vehicle or a single dose of arbaclofen were allowed to interact with two identical objects. After a delay, mice were tested by exposure to the two trained objects, one in its previous location, and one moved to a novel location within the testing arena. Time spent exploring each object during training (G) and testing (H) phases is shown. Data collected in the Crawley lab. Data were analyzed by 3-way ANOVA (genotype and treatment as within-subjects factors, location as within-subjects factor). n= 15, 17, 14, 12. **G.** Training phase: 2-way RMANOVA: No significant main effects of object ($F(1, 54) = 0.144$, $p =0.706$), genotype ($F(1,54) = 2.969$, $p = 0.0906$), treatment ($F(1,54) = 0.267$, $p =0.6072$), or significant interactions. **H.** Testing phase: 3-way RMANOVA: significant main effect of location ($F(1, 54) = 12.376$, $p = 8.9x10^{-4}$). No significant main effects of genotype ($F(1,54) = 0.949$, $p = 0.334$), treatment ($F(1,54) = 0.099$, $p = 0.754$), or significant interactions.

bioRxiv preprint doi: https://doi.org/10.1101/2023.05.01.538987; this version posted September 15, 2023. The copyright holder for this preprint (which was not certified by peer review) is the author/funder, who has granted bioRxiv a license to display the preprint in perpetuity. It is made available under aCC-BY 4.0 International license.

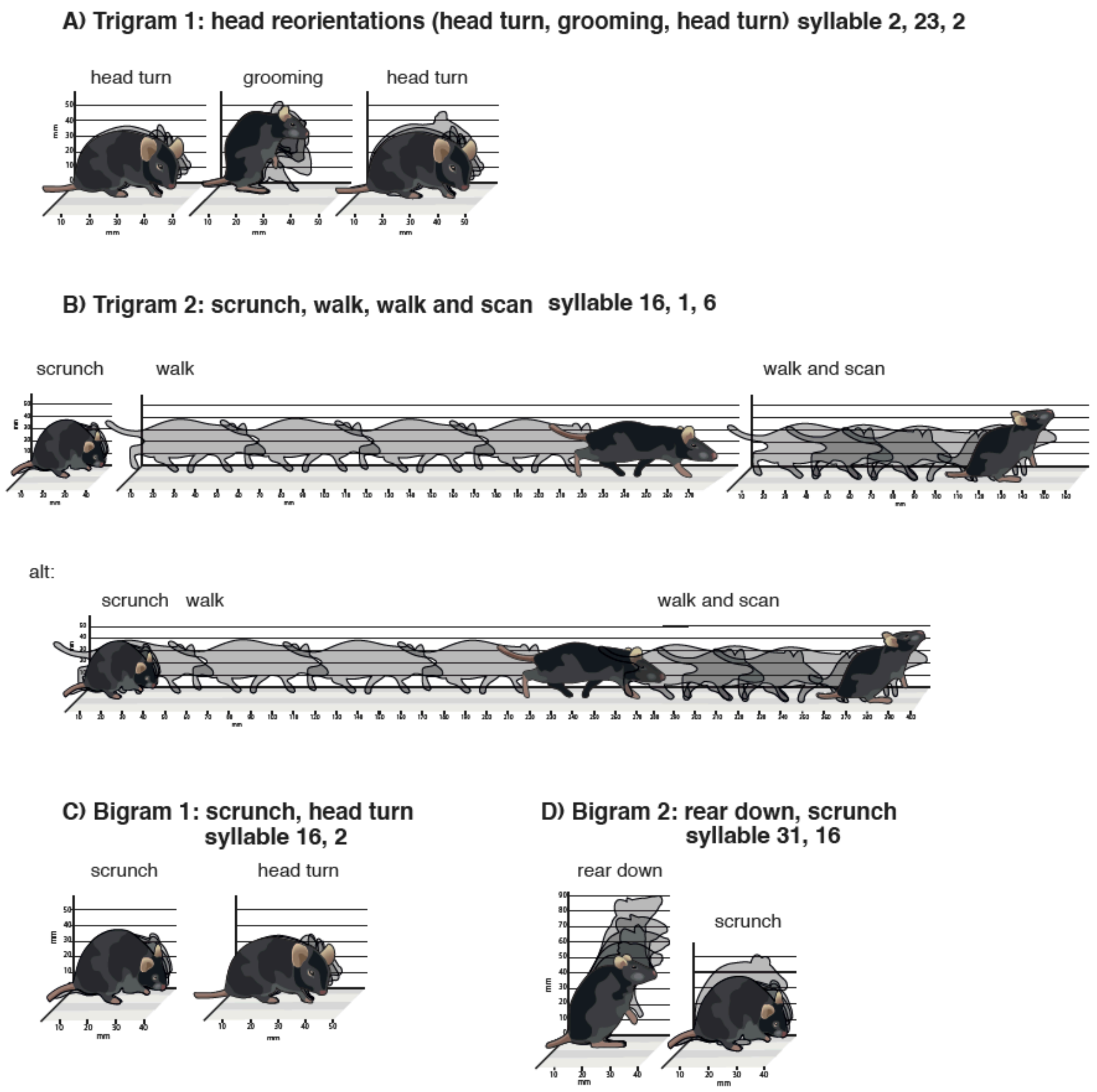

Figure S4. Artist's rendering of bigrams and trigrams. Figure credit: Sigrid Knemeyer, Scistories.

bioRxiv preprint doi: https://doi.org/10.1101/2023.05.01.538987; this version posted September 15, 2023. The copyright holder for this preprint (which was not certified by peer review) is the author/funder, who has granted bioRxiv a license to display the preprint in perpetuity. It is made available under aCC-BY 4.0 International license.

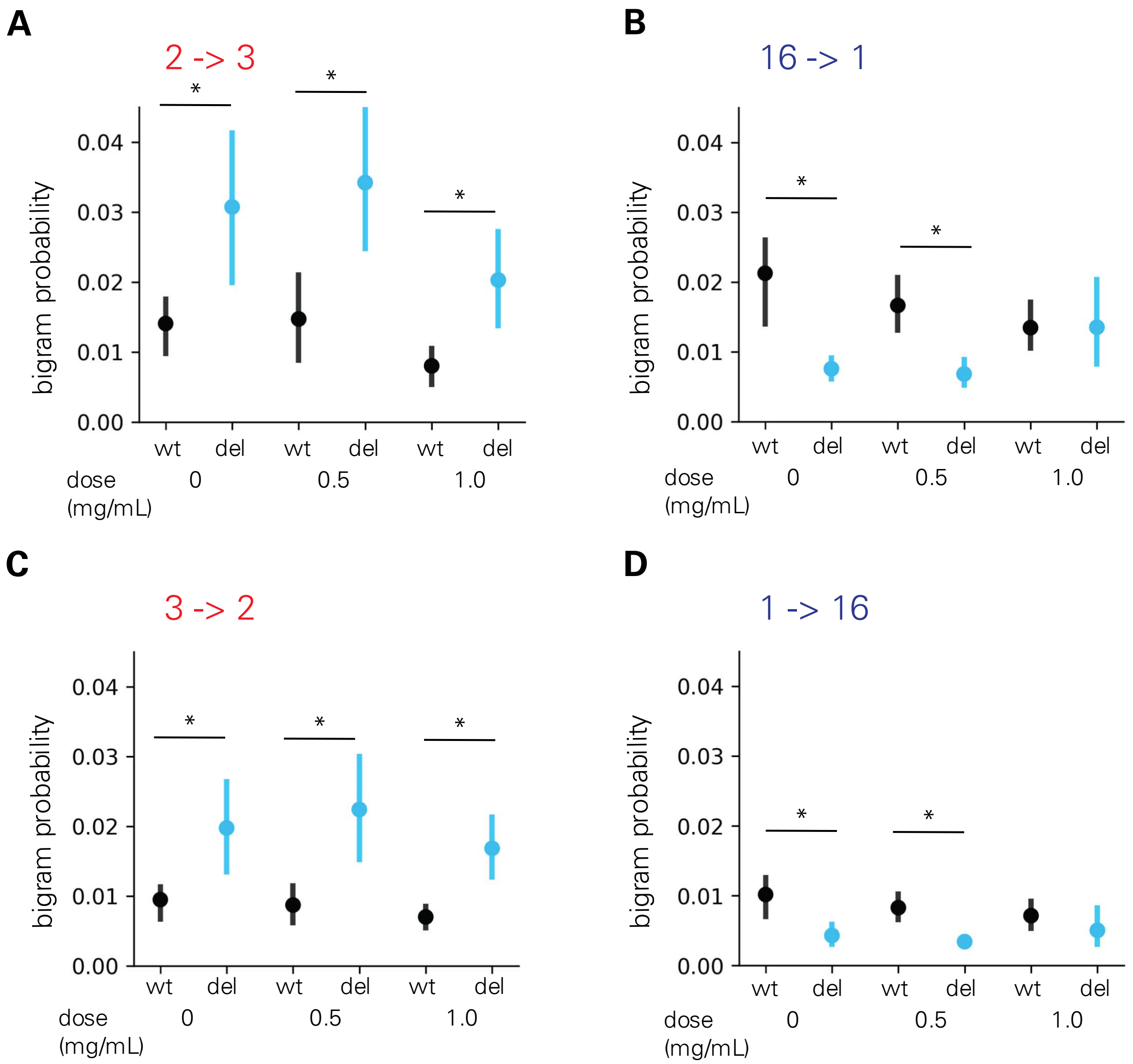

Figure S5. Differences in bigram probability in 16p11.2 Del4 deletion model mice are rescued by arbaclofen treatment. **A-D.** Data collected in the Datta lab. Example graphs showing probabilities of expressing individual bigrams across genotype and treatment groups. Error bars indicate 95% confidence intervals from 1000 bootstrapping and asterisks indicate non-overlap of confidence intervals between wildtype and 16p11.2 del for each condition. n= 15 wt/vehicle, 12 16p del/vehicle, 27 wt/0.5 mg/mL arbac, 14 16p del/0.5 mg/mL arbac, 18 wt/1.0 mg/mL arbac, 11 16p del/1.0 mg/mL arbac

bioRxiv preprint doi: https://doi.org/10.1101/2023.05.01.538987; this version posted September 15, 2023. The copyright holder for this preprint (which was not certified by peer review) is the author/funder, who has granted bioRxiv a license to display the preprint in perpetuity. It is made available under aCC-BY 4.0 International license.

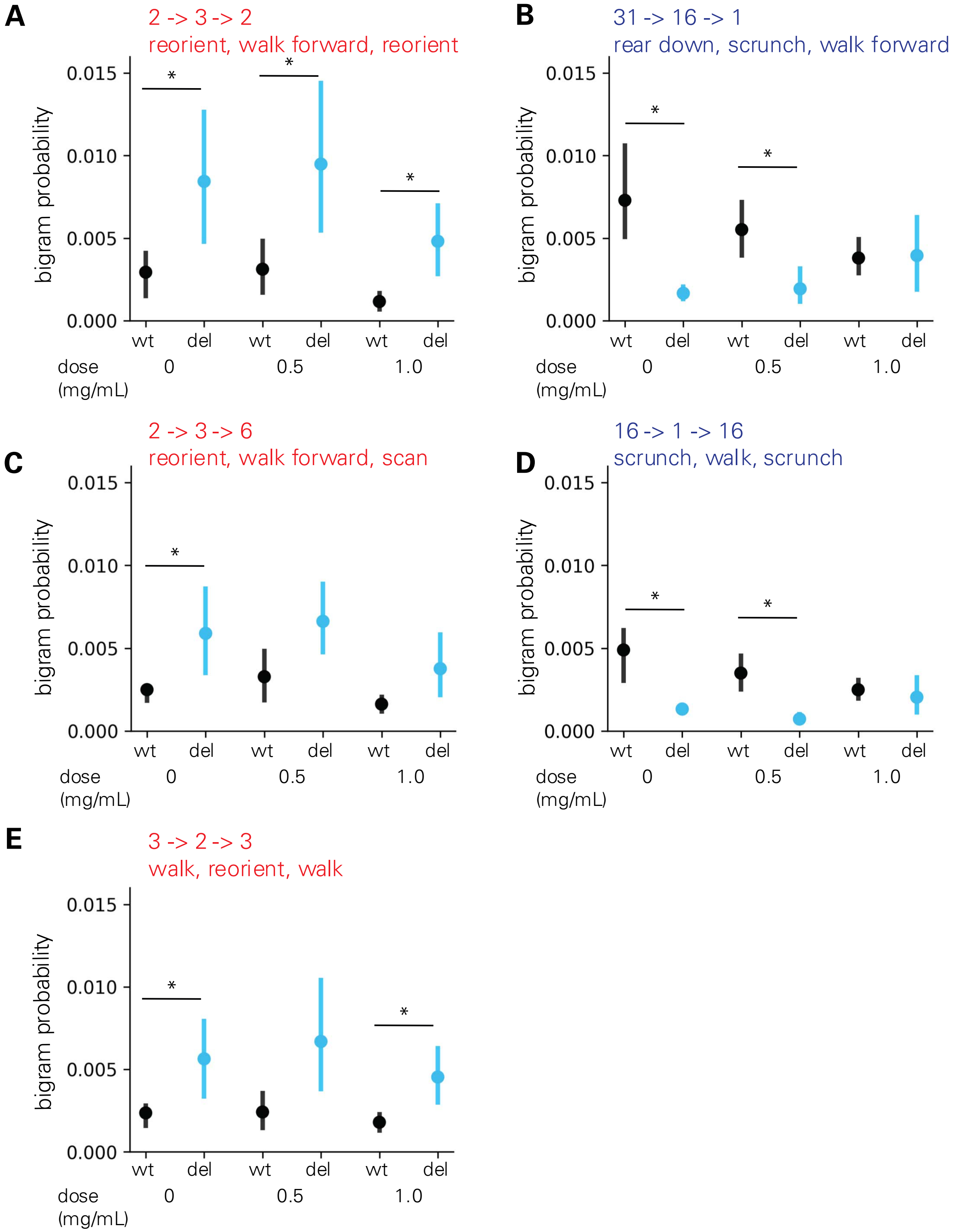

Figure S6. Differences in trigram probability in 16p11.2 Del4 deletion model mice are rescued by arbaclofen treatment. **A-E.** Data collected in the Datta lab. Example graphs showing probabilities of expressing individual trigrams across genotype and treatment groups. Error bars

bioRxiv preprint doi: https://doi.org/10.1101/2023.05.01.538987; this version posted September 15, 2023. The copyright holder for this preprint (which was not certified by peer review) is the author/funder, who has granted bioRxiv a license to display the preprint in perpetuity. It is made available under aCC-BY 4.0 International license.

indicate 95% confidence intervals from 1000 bootstrapping and asterisks indicate non-overlap of confidence intervals between wildtype and 16p11.2 deletion model mice for each condition. n= 15 wt/vehicle, 12 16p del/vehicle, 27 wt/0.5 mg/mL arbac, 14 16p del/0.5 mg/mL arbac, 18 wt/1.0 mg/mL arbac, 11 16p del/1.0 mg/mL arbac

bioRxiv preprint doi: https://doi.org/10.1101/2023.05.01.538987; this version posted September 15, 2023. The copyright holder for this preprint (which was not certified by peer review) is the author/funder, who has granted bioRxiv a license to display the preprint in perpetuity. It is made available under aCC-BY 4.0 International license.

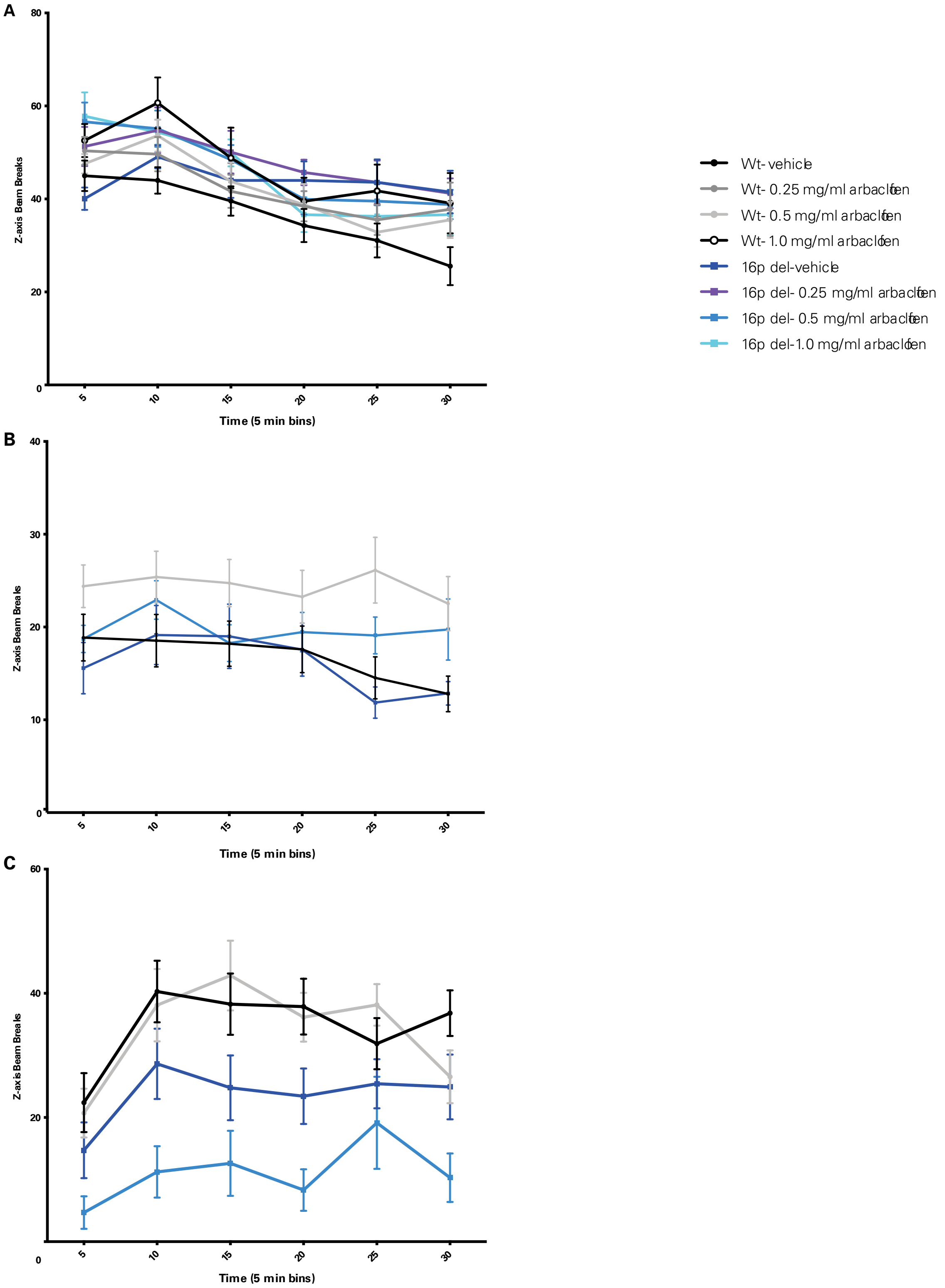

Figure S7. Inconsistent effects of arbaclofen on rearing behavior. Wildtype and 16p11.2 deletion model mice treated with one of 3 doses of arbaclofen were exposed to an open field for 30 min. Rearing counts (measured by infrared beam breaks in the z plane) are plotted in 5-

bioRxiv preprint doi: https://doi.org/10.1101/2023.05.01.538987; this version posted September 15, 2023. The copyright holder for this preprint (which was not certified by peer review) is the author/funder, who has granted bioRxiv a license to display the preprint in perpetuity. It is made available under aCC-BY 4.0 International license.

minute bins. Points are means with error bars indicating SEM. Data were analyzed with 3-way RMANOVA (genotype and treatment as between-subjects variables, time as within-subject variable).

A. Data were collected in the Herault lab (Del6 model). 3-way RMANOVA: main effect of time ($F(5,101) = 50.699$, $p<0.0001$), no significant main effect of genotype ($F(1, 101) = 2.026$, $p = 0.1577$), or treatment ($F(3, 101) = 1.154$, $p = 0.3312$). Significant time x treatment x genotype interaction ($F(15, 101) = 2.067$, $p = 0.0103$). n = 14, 14, 14, 12, 14, 14, 15, 11.

B. Data were collected in the Abel lab (Del4 model). 3-way RMANOVA: significant main effect of time ($F(5, 44) = 3.092$, $p=0.0102$), significant main effect of treatment ($F(1, 44) = 7.824$, $p=0.00762$), no significant main effect of genotype ($F(1, 44) = 1.044$, $p = 0.31245$). Significant time x treatment interaction ($F(5, 44) = 2.265$, $p=0.0491$). n = 15, 15, 7, 11.

C. Data were collected in the Crawley lab (Del1 model). 3-way RMANOVA: significant main effect of time ($F(5, 64) = 14.080$, $p = 1.87 \times 10^{-12}$), significant main effect of genotype ($F(1, 64) = 17.712$, $p = 8.18 \times 10^{-5}$), no significant main effect of treatment ($F(1, 64) = 2.037$, $p = 0.158$). Significant time x treatment interaction ($F(5, 64) = 2.420$, $p=0.0357$). n = 20, 21, 14, 13.

bioRxiv preprint doi: https://doi.org/10.1101/2023.05.01.538987; this version posted September 15, 2023. The copyright holder for this preprint (which was not certified by peer review) is the author/funder, who has granted bioRxiv a license to display the preprint in perpetuity. It is made available under aCC-BY 4.0 International license.

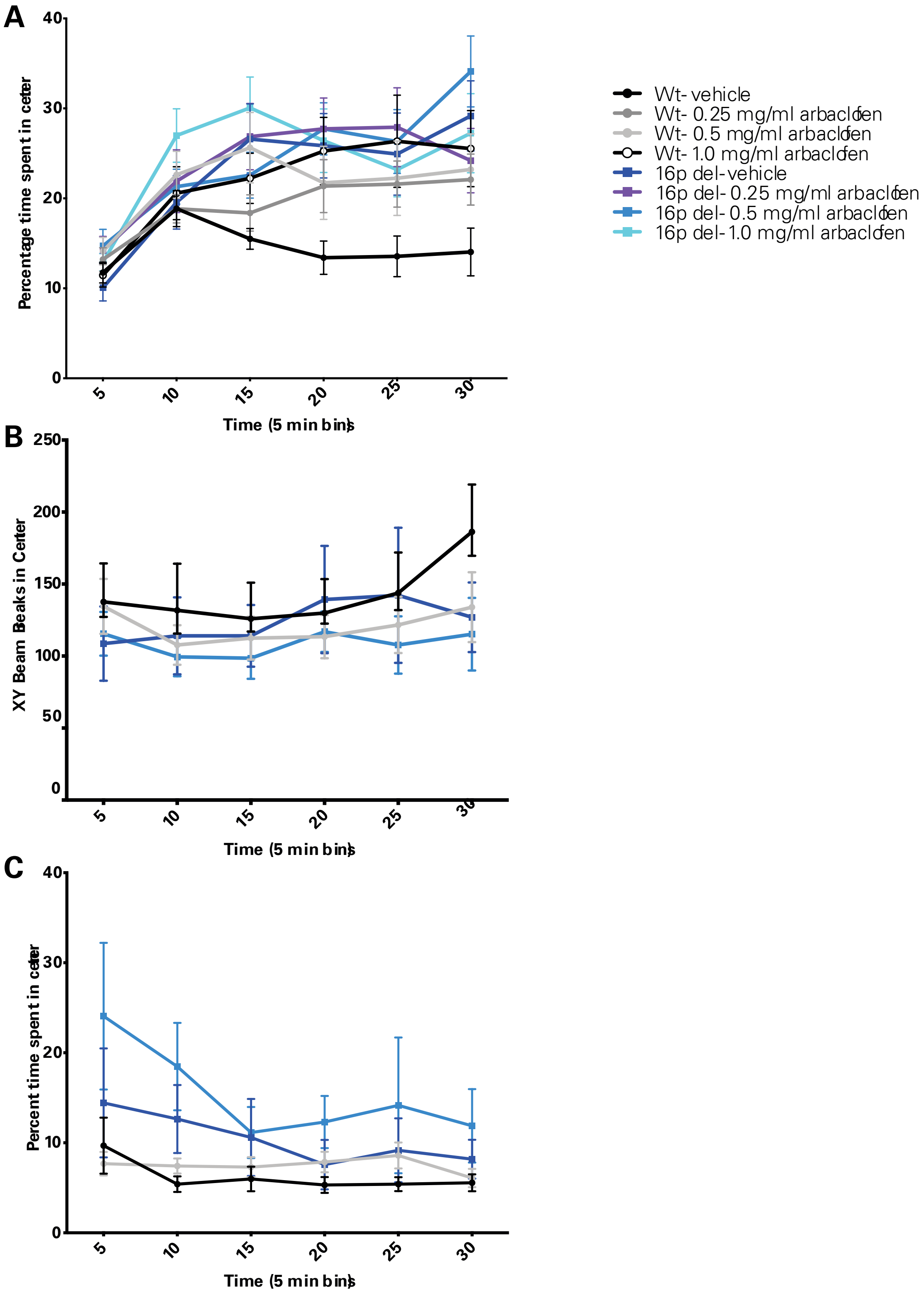

Figure S8. Inconsistent effects of arbaclofen on behavior in the center of the open field. Wildtype and 16p11.2 deletion model mice treated with one of 3 doses of arbaclofen were exposed to an open field for 30 min. Distance traveled in the center of the open field (measured

bioRxiv preprint doi: https://doi.org/10.1101/2023.05.01.538987; this version posted September 15, 2023. The copyright holder for this preprint (which was not certified by peer review) is the author/funder, who has granted bioRxiv a license to display the preprint in perpetuity. It is made available under aCC-BY 4.0 International license.

as infrared beam breaks in the x and y planes within a predefined square in the center of the larger arena) or percentage of time spent in the center of the open field is plotted in 5-minute bins. Points are means with error bars indicating SEM. Data were analyzed with 3-way RMANOVA (genotype and treatment as between-subjects variables, time as within-subject variable).

A. Data were collected in the Herault lab (Del6 model). Percent time spent in the center of the open field is plotted. 3-way RMANOVA: significant main effect of time ($F(5, 101) = 33.454$, $p < 0.0001$), significant main effect of genotype ($F(1,101) = 6.979$, $p = 0.0096$), no significant main effect of treatment ($F(3,101) = 1.650$, $p = 0.1827$), significant time x genotype interaction ($F(5,101)=3.211$, $p =0.0073$), significant time x genotype x treatment interaction ($F(15, 101) = 2.404$, $p = 0.0022$). n = 14, 14, 14, 12, 14, 14, 15, 12.

B. Data were collected in the Abel lab (Del4 model). Distance traveled in the center of the open field is plotted. 3-way RMANOVA: significant main effect of time ($F(5, 45) = 4.082$, $p=0.00144$). No significant main effect of treatment ($F(1, 45) = 1.087$, $p = 0.303$), genotype ($F(1, 45) = 0.802$, $p = 0.375$), or significant interactions. n = 16, 14, 8, 11.

C. Data were collected in the Crawley lab (Del1 model). Percent time spent in the center of the open field is plotted. 3-way RMANOVA: significant main effect of time ($F(5,61) = 2.559$, $p=0.0275$), significant main effect of genotype ($F(1,61) = 9.169$, $p = 0.0036$), no significant main effect of treatment ($F(1,61) = 1.697$, $p = 0.1976$). No significant interactions. n = 21, 20, 14, 12.

bioRxiv preprint doi: https://doi.org/10.1101/2023.05.01.538987; this version posted September 15, 2023. The copyright holder for this preprint (which was not certified by peer review) is the author/funder, who has granted bioRxiv a license to display the preprint in perpetuity. It is made available under aCC-BY 4.0 International license.

A

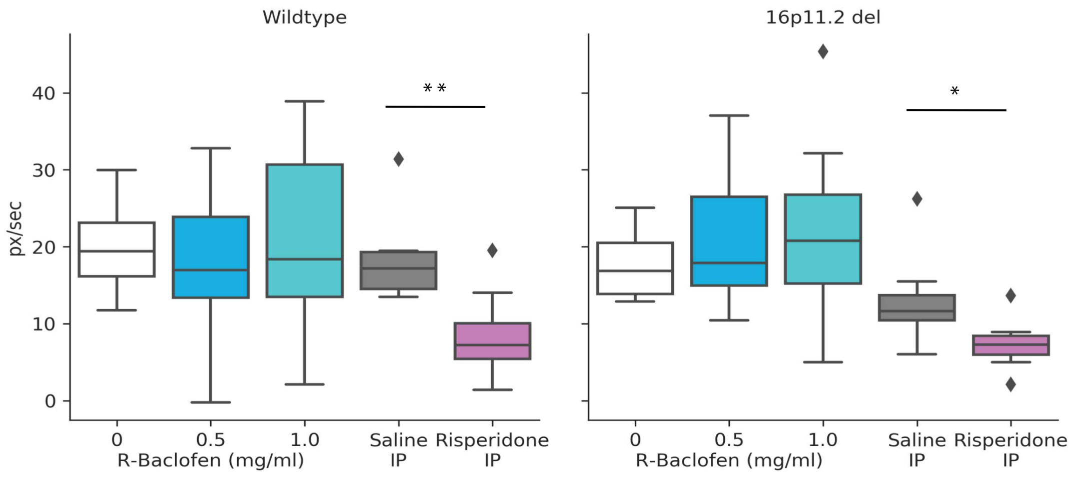

B

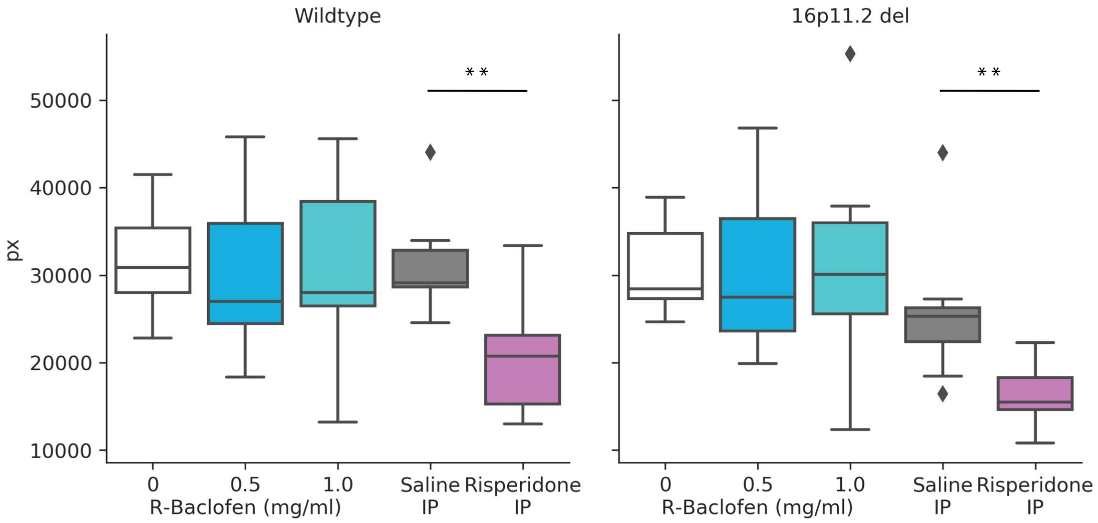

Figure S9. Risperidone reduces velocity of movement and distance traveled regardless of genotype, whereas arbaclofen does not affect these measures.

Data were collected in the Datta lab (Del4 model). Graphs are box and whisker plots; whiskers are maximum and minimum, boxes are interquartile range, and midline is median. Wildtype: n= 15, 27, 18, 6, 13. 16p11.2 del: n= 12, 14, 11, 11, 8. *$p < 0.05$ vs. vehicle; **$p < 0.01$ vs. vehicle.

A. Mean angular velocity over the entire recording session was measured from videos. Mann-Whitney U-test was performed to examine the difference between control and drug groups (separate analysis for arbaclofen and risperidone vs. control). Wildtype: 0.5 mg/mL arbaclofen vs. vehicle U = 171, p = 0.4158. 1.0 mg/mL arbaclofen vs. vehicle: U = 131, p = 0.8993. Risperidone vs. vehicle: U = 6, p =0.0044. 16p11.2 del: 0.5 mg/mL arbaclofen vs. vehicle U = 74,

bioRxiv preprint doi: https://doi.org/10.1101/2023.05.01.538987; this version posted September 15, 2023. The copyright holder for this preprint (which was not certified by peer review) is the author/funder, who has granted bioRxiv a license to display the preprint in perpetuity. It is made available under aCC-BY 4.0 International license.

p = 0.6251. 1.0 mg/mL arbaclofen vs. vehicle: U = 47, p = 0.2549. Risperidone vs. vehicle: U = 15, p =0.0186.

B. Total distance traveled over the entire recording session was measured from videos. Mann-Whitney U-test was performed to examine the difference between control and drug groups (separate analysis for arbaclofen and risperidone vs. control). Wildtype: 0.5 mg/mL arbaclofen vs. vehicle U = 166, p = 0.3447. 1.0 mg/mL arbaclofen vs. vehicle: U = 117, p = 0.8993. Risperidone vs. vehicle: U = 8, p =0.0075. 16p11.2 del: 0.5 mg/mL arbaclofen vs. vehicle U = 70, p = 0.4875. 1.0 mg/mL arbaclofen vs. vehicle: U = 66, p = 0.9755. Risperidone vs. vehicle: U = 6, p =0.0020.
